# Draft Genomes of 75 *Vibrio* Isolates from Retail Mollusks Collected in Canada between 2014 and 2018

**DOI:** 10.1128/mra.00748-22

**Published:** 2022-11-09

**Authors:** Januana S. Teixeira, Forest Dussault, Emily Hoover, Julie A. Shay, Kelly Weedmark, Swapan K. Banerjee

**Affiliations:** a Bureau of Microbial Hazards, Health Canada, Ottawa, Ontario, Canada; b Bureau of Food Surveillance and Science Integration, Health Canada, Ottawa, Ontario, Canada; Indiana University, Bloomington

## Abstract

*Vibrio* spp. isolated from fresh retail mollusk samples were selected for sequencing based on their antimicrobial resistance burden. The *de novo* genomes include those for Vibrio alginolyticus (*n* = 48), *V. diabolicus* (*n* = 15), V. parahaemolyticus (*n* = 3), V. cholerae (*n* = 2), *V. metoecus* (*n* = 1), V. vulnificus (*n* = 1), *V. fluvialis* (*n* = 1), and unidentified *Vibrio* spp. (*n* = 4).

## ANNOUNCEMENT

*Vibrio* spp. represent a diverse group of Gram-negative human bacterial pathogens responsible for the majority of foodborne illnesses associated with seafood (1, 2). Illness resulting from these bacteria arises primarily from consuming raw or undercooked mollusks contaminated with pathogenic *Vibrio* during warmer months of the year (1, 3). There is growing concern that marine pathogens, including *Vibrio* spp., which are exposed to antimicrobial resistant (AMR) bacteria, may themselves become resistant to standard antibiotic treatment (4). The present study sequenced the genomes of a subset (*n* = 75) of *Vibrio* isolates from fresh retail mollusks available in Canada (2014 to 2018) based on AMR profiles.

Single colonies from fresh retail mollusk samples (*n* = 45) were isolated as described (5). Briefly, 10 to 20 shucked mollusks per sample were homogenized in a blender; a total of 50 g of homogenate was mixed with 450 mL alkaline peptone water (1% Bacto-peptone and 2% NaCl [pH 8.5]) and equilibrated 1 h at 21°C before overnight enrichment at 35°C. Selective agars (thiosulfate-citrate-bile salts-sucrose [TCBS], CHROMagar, and mCC10 or CC400) were used for both pre-enrichment (100 μL) and postenrichment (10 μL) plating. After a 35°C overnight incubation, presumptive *Vibrio* colonies were grown at 35°C overnight on tryptic soy agar with 2% NaCl (TSA-2N) (Difco BD, Franklin Lakes, NJ) for molecular/biochemical *Vibrio* confirmation (5) and −80°C stock preparation (10 μL loopful/Microbank vial) (VWR, Mississauga, Ontario, Canada). The AMR phenotype to 23 antibiotics (Oxoid Ltd., Basingstoke, UK) was determined for isolates using the Kirby-Bauer disk diffusion method and published guidelines (6–8); a subset of isolates with AMR profiles ranging from no resistance to multidrug resistance was selected for sequencing (Table 1).

**TABLE 1:** Metadata and genome assembly metrics for 75 *Vibrio* sp. isolates collected from fresh retail mollusks available in Canada from 2014 to 2018

Isolate	Mollusk type	Yr	Retail location^*a*^	Taxonomic ID^*b*^ (Mash)	AMR profile^*c*^	Genome size (bp)	GC content (%)	Coverage (×)	No. of contigs	No. of reads	*N*_50_ (bp)	No. of genes	BioSample accession	Assembly accession	GenBank accession	SRA accession
RM-6-1	Mussel	2014	PEI	V. alginolyticus	AMP, PIP, S	5,139,382	44.6	57	65	1,086,716	225,218	4,694	SAMN28767208	GCA_024744935.1	JAMQVD000000000	SRR19544305
RM-11-1	Oyster	2014	PEI	V. alginolyticus	AMP (KF, E)	5,237,869	44.6	58	76	1,123,262	446,189	4,876	SAMN28767209	GCA_024744915.1	JAMQVE000000000	SRR19544304
RM-14-4	Mussel	2014	PEI	V. alginolyticus	AMP, PIP, KF	5,227,775	44.6	63	71	1,188,912	249,827	4,832	SAMN28767210	GCA_024744895.1	JAMQVF000000000	SRR19544293
RM-15-5	Oyster	2014	BC	V. alginolyticus	AMP, PIP, KF	5,126,382	44.9	70	53	1,035,206	405,367	4,635	SAMN28767211	GCA_024744855.1	JAMQVG000000000	SRR19544282
RM-15-6	Oyster	2014	BC	V. alginolyticus	AMP, PIP, KF	5,118,101	44.5	63	65	1,335,228	221,239	4,703	SAMN28767212	GCA_024744835.1	JAMQVH000000000	SRR19544247
RM-15-9	Oyster	2014	BC	V. alginolyticus	AMP, PIP, KF	5,128,533	44.5	50	50	1,246,452	379,764	4,707	SAMN28767213	GCA_024744815.1	JAMQVI000000000	SRR19544236
RM-15-10	Oyster	2014	BC	*V. diabolicus*	AMP, PIP, OX	5,054,262	44.5	56	41	908,836	347,902	4,702	SAMN28767214	GCA_024744845.1	JAMQVJ000000000	SRR19544273
RM-16-10	Mussel	2014	PEI	V. alginolyticus	AMP, PIP, KF	5,271,750	44.6	58	40	1,112,442	385,123	4,788	SAMN28767215	GCA_024744795.1	JAMQVK000000000	SRR19544262
RM-16-11	Mussel	2014	PEI	V. alginolyticus	AMP, PIP, KF	5,178,540	44.6	80	51	1,574,650	290,945	4,786	SAMN28767216	GCA_024744755.1	JAMQVL000000000	SRR19544256
RM-17-4	Clam	2014	BC	V. alginolyticus	AMP, PIP, KF	5,142,659	44.6	49	54	917,938	364,175	4,672	SAMN28767217	GCA_024744735.1	JAMQVM000000000	SRR19544255
RM-17-5	Clam	2014	BC	V. alginolyticus	AMP, PIP, KF	5,138,442	44.6	45	53	866,500	290,122	4,665	SAMN28767218	GCA_024744775.1	JAMQVN000000000	SRR19544303
RM-18-1	Oyster	2014	PEI	V. alginolyticus	AMP, PIP, KF	5,218,565	44.6	53	56	1,028,156	248,131	4,844	SAMN28767219	GCA_024744715.1	JAMQVO000000000	SRR19544302
RM-18-3	Oyster	2014	PEI	V. alginolyticus	AMP, PIP, KF	5,217,942	44.6	55	48	1,027,504	442,486	4,833	SAMN28767220	GCA_024744695.1	JAMQVP000000000	SRR19544301
RM-20-4	Mussel	2014	PEI	V. alginolyticus	AMP, PIP, KF	5,513,473	44.6	44	53	882,848	422,698	5,141	SAMN28767221	GCA_024744655.1	JAMQVQ000000000	SRR19544300
RM-30-9	Clam	2015	MB	*V. diabolicus*	AMP, SF, KF, OX	5,051,443	44.7	49	54	881,366	336,942	4,727	SAMN28767222	GCA_024744635.1	JAMQVR000000000	SRR19544299
RM-31-4	Mussel	2015	PEI	V. alginolyticus	AMP, PIP, KF	5,269,051	44.6	42	52	790,774	230,154	4,873	SAMN28767223	GCA_024744615.1	JAMQVS000000000	SRR19544298
RM-32-1	Oyster	2015	BC	V. alginolyticus	AMP (PIP, KF, E)	5,264,157	44.5	113	62	2,214,222	240,981	4,869	SAMN28767224	GCA_024744645.1	JAMQVT000000000	SRR19544297
RM-32-14	Oyster	2015	BC	V. alginolyticus	AMP, PIP, KF, SF	5,287,414	44.5	72	97	1,402,938	159,185	4,867	SAMN28767225	GCA_024744595.1	JAMQVU000000000	SRR19544296
RM-33-1	Mussel	2015	BC	V. alginolyticus	AMP, PIP, KF	5,305,958	44.5	42	46	816,680	440,386	4,867	SAMN28767226	GCA_024744575.1	JAMQVV000000000	SRR19544295
RM-35-7	Mussel	2015	BC	V. alginolyticus	AMP, PIP, KF	5,252,659	44.5	39	57	733,334	315,487	4,824	SAMN28767227	GCA_024744545.1	JAMQVW000000000	SRR19544294
RM-35-9	Mussel	2015	BC	*V. diabolicus*	AMP, KF, OX	5,064,064	44.8	34	47	596,670	428,425	4,648	SAMN28767228	GCA_024744525.1	JAMQVX000000000	SRR19544292
RM-36-7	Clam	2015	BC	V. alginolyticus	AMP, PIP, KF	5,145,530	44.4	35	72	656,786	171,080	4,655	SAMN28767229	GCA_024744515.1	JAMQVY000000000	SRR19544291
RM-38-7	Clam	2015	BC	V. alginolyticus	AMP, PIP, KF	5,263,704	44.6	60	38	1,158,522	286,398	4853	SAMN28767230	GCA_024744495.1	JAMQVZ000000000	SRR19544290
RM-39-4	Clam	2015	BC	V. alginolyticus	AMP, PIP, KF	5,239,905	44.6	76	50	1,659,760	368,966	4,879	SAMN28767231	GCA_024744475.1	JAMQWA000000000	SRR19544289
RM-39-6	Clam	2015	BC	V. alginolyticus	AMP, PIP, KF	5,289,632	44.5	84	69	1,703,254	196,839	4,864	SAMN28767232	GCA_024744455.1	JAMQWB000000000	SRR19544288
RM-41-2A	Oyster	2015	BC	*Vibrio* spp.	AMP, PIP, OX	5,203,605	44.8	82	108	1,572,494	116,867	4,788	SAMN28767233	GCA_024744435.1	JAMQWC000000000	SRR19544287
RM-41-2B	Oyster	2015	BC	*Vibrio* spp.	AMP, PIP, KF, OX	5,213,084	44.8	49	57	925,380	276,740	4,780	SAMN28767234	GCA_024744415.1	JAMQWD000000000	SRR19544286
RM-41-6	Oyster	2015	BC	V. alginolyticus	AMP, KF (PIP, CTX, E, ENO)	5,590,557	44.4	110	87	2,453,644	193,719	5,245	SAMN28767235	GCA_024744395.1	JAMQWE000000000	SRR19544285
RM-42-4	Clam	2015	BC	*V. diabolicus*	AMP, PIP, OX	5,296,987	44.7	56	68	1,088,760	223,223	4,926	SAMN28767236	GCA_024744375.1	JAMQWF000000000	SRR19544284
RM-44-2	Oyster	2015	BC	V. alginolyticus	AMP, PIP, KF	5,352,218	44.6	51	85	998,750	178,940	5,005	SAMN28767237	GCA_024744355.1	JAMQWG000000000	SRR19544283
RM-44-3	Oyster	2015	BC	*Vibrio* spp.	AMP, PIP, KF, SF, OX	5,203,331	44.8	85	131	1,630,898	99,981	4,802	SAMN28767238	GCA_024744335.1	JAMQWH000000000	SRR19544281
RM-46-1	Clam	2015	BC	V. alginolyticus	AMP, PIP, KF	5,146,619	44.6	42	66	773,280	219,393	4,795	SAMN28767239	GCA_024744305.1	JAMQWI000000000	SRR19544280
RM-47-7	Mussel	2015	BC	V. alginolyticus	AMP, PIP, KF, SF, OX	5,234,538	44.6	60	92	1,159,622	112,517	4,798	SAMN28767240	GCA_024744285.1	JAMQWJ000000000	SRR19544279
RM-48-6	Mussel	2015	PEI	V. alginolyticus	AMP, PIP, KF	5,139,195	44.5	97	83	1,931,742	165,239	4,728	SAMN28767241	GCA_024744275.1	JAMQWK000000000	SRR19544254
RM-49-4	Oyster	2015	NB	V. alginolyticus	AMP, PIP, OX	5,290,784	44.5	29	97	565,670	109,439	4,890	SAMN28767242	GCA_024744255.1	JAMQWL000000000	SRR19544253
RM-49-5	Oyster	2015	NB	V. alginolyticus	AMP, PIP, OT	5,138,153	44.6	38	109	750,646	97,040	4,680	SAMN28767243	GCA_024744235.1	JAMQWM000000000	SRR19544252
RM-49-6	Oyster	2015	NB	V. alginolyticus	AMP, PIP, KF, OX	5,174,447	44.5	64	73	1,232,382	186,688	4,816	SAMN28767244	GCA_024744215.1	JAMQWN000000000	SRR19544251
RM-50-2	Mussel	2015	BC	*V. diabolicus*	AMP, PIP, OX	5,120,376	44.8	51	160	1,416,472	68,872	4,834	SAMN28767245	GCA_024744195.1	JAMQWO000000000	SRR19544250
RM-50-3	Mussel	2015	BC	*V. diabolicus*	AMP, PIP, OX	5,139,456	44.8	34	91	953,844	150,209	4,840	SAMN28767246	GCA_024744175.1	JAMQWP000000000	SRR19544249
RM-51-4	Clam	2015	BC	V. alginolyticus	AMP, PIP, KF	5,335,949	44.5	36	92	715,712	161,255	4,946	SAMN28767247	GCA_024744155.1	JAMQWQ000000000	SRR19544248
RM-51-5	Clam	2015	BC	V. alginolyticus	AMP, PIP, KF	5,336,320	44.5	55	68	1,183,996	215,337	4,949	SAMN28767248	GCA_024744105.1	JAMQWR000000000	SRR19544246
RM-53-1	Oyster	2015	QC	*V. diabolicus*	AMP, PIP, KF, SF, OX	5,179,548	44.8	46	108	1,007,130	87,078	4,759	SAMN28767249	GCA_024744135.1	JAMQWS000000000	SRR19544245
RM-53-2	Oyster	2015	QC	*V. diabolicus*	AMP, PIP, KF, OX	5,184,243	44.7	39	75	721,366	121,173	4,770	SAMN28767250	GCA_024744095.1	JAMQWT000000000	SRR19544244
RM-54-2	Oyster	2015	NB	*V. diabolicus*	AMP, PIP, OX	5,043,200	44.8	67	51	1,313,972	230,783	4,620	SAMN28767251	GCA_024744055.1	JAMQWU000000000	SRR19544243
RM-54-3	Oyster	2015	NB	*V. diabolicus*	AMP, PIP, KF, OX	5,051,438	44.8	78	45	1,552,654	389,118	4,623	SAMN28767252	GCA_024744075.1	JAMQWV000000000	SRR19544242
RM-55-3	Oyster	2015	QC	*V. diabolicus*	AMP, PIP, KF, SF, OX	5,102,463	44.9	74	41	1,349,826	363,436	4,781	SAMN28767253	GCA_024744025.1	JAMQWW000000000	SRR19544241
RM-58-1	Mussel	2015	NL	V. alginolyticus	AMP, PIP, KF, SF	5,195,584	44.6	154	45	2,987,352	285,387	4,737	SAMN28767254	GCA_024744015.1	JAMQWX000000000	SRR19544240
RM-58-2	Mussel	2015	NL	V. alginolyticus	AMP, PIP, KF, PB, OX	5,194,433	44.6	48	52	1,140,158	346,030	4,746	SAMN28767255	GCA_024743995.1	JAMQWY000000000	SRR19544239
RM-59-1	Oyster	2015	PEI	V. alginolyticus	AMP, PIP, KF, SF	5,223,928	44.6	47	224	929,392	58,091	4,830	SAMN28767256	GCA_024743975.1	JAMQWZ000000000	SRR19544238
RM-59-2	Oyster	2015	PEI	V. alginolyticus	AMP, PIP, KF, SF	5,223,283	44.6	42	209	816,194	52,452	4,823	SAMN28767257	GCA_024743955.1	JAMQXA000000000	SRR19544237
RM-62-1	Oyster	2015	QC	V. alginolyticus	AMP, PIP, KF	5,266,495	44.6	41	93	813,524	106,402	4,787	SAMN28767258	GCA_024743935.1	JAMQXB000000000	SRR19544235
RM-62-3	Oyster	2015	QC	V. alginolyticus	AMP, PIP, KF	5,264,540	44.6	124	50	2,500,242	30,3236	4,782	SAMN28767259	GCA_024743875.1	JAMQXC000000000	SRR19544234
RM-63-1	Mussel	2015	PEI	V. alginolyticus	AMP, PIP, KF, SF	5,198,355	44.7	30	243	549,986	44,441	4,835	SAMN28767260	GCA_024743895.1	JAMQXD000000000	SRR19544233
RM-63-2	Mussel	2015	PEI	V. alginolyticus	AMP, PIP, KF, SF	5,244,519	44.6	55	81	1,031,798	149,576	4,854	SAMN28767261	GCA_024743915.1	JAMQXE000000000	SRR19544232
RM-65-3	Clam	2015	NB	*V. diabolicus*	AMP, KF, OX	5,034,284	44.8	43	65	772,454	146,395	4,605	SAMN28767262	GCA_024743855.1	JAMQXF000000000	SRR19544231
RM-67-1	Mussel	2015	PEI	V. alginolyticus	AMP, PIP, KF	5,244,311	44.6	36	289	1,051,714	43,360	4,897	SAMN28767263	GCA_024743835.1	JAMQXG000000000	SRR19544278
RM-67-3	Mussel	2015	PEI	V. alginolyticus	AMP, PIP, KF	5,276,861	44.5	59	157	1,115,426	85,282	4,912	SAMN28767264	GCA_024743805.1	JAMQXH000000000	SRR19544277
RM-69-4	Oyster	2015	NB	*Vibrio* spp.^*d*^	AMP, KF, IPM, DOR (ETP, E, ENO, OT)	4,036,806	44.5	317	98	4,749,982	237,730	3,687	SAMN28767265	GCA_024743775.1	JAMQXI000000000	SRR19544276
RM-69-5	Oyster	2015	NB	V. alginolyticus	AMP, PIP, KF	5,177,717	44.6	58	87	1,128,036	131,966	4,806	SAMN28767266	GCA_024743795.1	JAMQXJ000000000	SRR19544275
RM-69-8	Oyster	2015	NB	V. alginolyticus	AMP, PIP, KF	5,178,556	44.6	46	85	871,556	131,184	4,808	SAMN28767267	GCA_024743745.1	JAMQXK000000000	SRR19544274
RM-75-1	Oyster	2015	NB	*V. diabolicus*	AMP, PIP, KF, OX	5,211,380	44.8	57	119	1,067,426	85,122	4,846	SAMN28767268	GCA_024743735.1	JAMQXL000000000	SRR19544272
RM-75-2	Oyster	2015	NB	*V. diabolicus*	AMP, KF, OX	5,220,945	44.8	47	64	934,704	262,542	4,834	SAMN28767269	GCA_024743715.1	JAMQXM000000000	SRR19544271
RM-80-5B2	Oyster	2016	NB	*V. fluvialis*	AMP, PIP, KF, CTX, CEF (ETP, IMP, SF, E, OT)	4,848,434	49.7	86	58	1,581,876	218,439	4,575	SAMN28767270	GCA_024743695.1	JAMQXN000000000	SRR19544270
RM-85-1	Mussel	2016	BC	V. alginolyticus	AMP, PIP, KF	5,420,155	44.6	54	65	1,054,496	301,057	5,064	SAMN28767271	GCA_024743675.1	JAMQXO000000000	SRR19544269
RM-86-11	Oyster	2016	PEI	V. parahaemolyticus	AMP, PIP, KF	5,019,268	45.3	70	30	1,297,124	371,872	4,618	SAMN28767272	GCA_024743635.1	JAMQXP000000000	SRR19544268
RM-89-5	Oyster	2016	BC	V. alginolyticus	AMP, PIP, KF	5,260,117	44.6	38	136	1,478,570	89,166	4,901	SAMN28767273	GCA_024743655.1	JAMQXQ000000000	SRR19544267
RM-89-6	Oyster	2016	BC	V. parahaemolyticus	AMP, PIP, KF	5,268,074	45.2	45	54	850,322	336,059	4,891	SAMN28767274	GCA_024743615.1	JAMQXR000000000	SRR19544266
RM-92-1	Mussel	2016	PEI	V. alginolyticus	AMP, KF	5,371,879	44.6	61	71	1,165,112	196,890	4,897	SAMN28767275	GCA_024743565.1	JAMQXS000000000	SRR19544265
RM-92-2	Mussel	2016	PEI	V. alginolyticus	AMP, KF (PIP, E, OT)	5,375,628	44.6	31	80	596,688	138,897	4,908	SAMN28767276	GCA_024743595.1	JAMQXT000000000	SRR19544264
RM-160-1	Mussel	2018	NS	V. cholerae	(DOR, IPM)	4,081,390	47.6	177	50	2,741,848	648,558	3,750	SAMN28767277	GCA_024743535.1	JAMQXU000000000	SRR19544263
RM-195-1	No data	2018	NS	V. cholerae	NONE	3,994,309	47.6	85	77	1,295,286	245,400	3,680	SAMN28767278	GCA_024743545.1	JAMQXV000000000	SRR19544261
RM-195-2	No data	2018	NS	*V. metoecus*	AMP, PIP, S	3,995,805	47.0	106	109	1,536,338	77,829	3,776	SAMN28767279	GCA_024743495.1	JAMQXW000000000	SRR19544260
RM-195-3	No data	2018	NS	V. vulnificus	S, SXT, SF, OT (TE)	5,205,873	46.6	69	63	1,337,300	414,924	4,744	SAMN28767280	GCA_024743455.1	JAMQXX000000000	SRR19544259
RM-211-4	Mussel	2018	NS	V. parahaemolyticus	S, SXT, SF, OT (PB, TE)	4,922,914	45.4	59	67	1,096,816	203,583	4,564	SAMN28767281	GCA_024743475.1	JAMQXY000000000	SRR19544258
RM-214-3	No data	2018	NS	*V. diabolicus*	AMP, PIP, KF, CTX, CEF (E)	5,342,114	44.9	115	143	2,270,098	86,108	4,975	SAMN28767282	GCA_024743515.1	JAMQXZ000000000	SRR19544257

a Canadian provinces: BC, British Columbia; MB, Manitoba; NB, New Brunswick; NL, Newfoundland; NS, Nova Scotia; PEI, Prince Edward Island; QC, Québec.

b ID, identifier.

c Antibiotics: AMP, ampicillin; CEF, ceftiofur; CTX, cefotaxime; C, chloramphenicol; CIP, ciprofloxacin; DOR, doripenem; ETP, ertapenem; E, erythromycin; ENO, enrofloxacin; GN, gentamicin; IPM, imipenem; K, kanamycin; KF, cephalothin; MEM, meropenem; NOR, norfloxacin; OT, oxytetracycline; OX, oxolinic acid; PIP, piperacillin; PB, polymyxin B; S, streptomycin; SF, sulfisoxazole; SXT, sulphamethoxazole-trimethoprim; TE, tetracycline. Brackets, intermediate resistance; NONE, no resistance.

d *V. anguillarum*/*V. ordalii*.

For sequencing, stocks were streaked onto TSA-2N and incubated at 35°C overnight. Single-colony inoculates (5 mL tryptic soy broth with 2% NaCl [TSB-2N]) were grown for 24 h at 35°C, and DNA was extracted using the Maxwell 16 SEV cell DNA purification kit (Promega, Madison, WI, USA). Paired-end Illumina whole-genome sequencing (WGS) was performed using indexed NexteraXT libraries run on a MiSeq instrument (2 × 300-bp cycles, v3 chemistry) according to the manufacturer (Illumina, San Diego, CA). Reads were trimmed to remove adapters, quality filtered, and error corrected (BBMap v38.26 [BBDuk and Tadpole]) (https://sourceforge.net/projects/bbmap) for *de novo* assembly (SKESA v2.3 and Pilon v1.22) (9), gene prediction (NCBI PGAP v6.1; best-placed reference protein set; GeneMarkS-2+) (10), and metric reporting (QUAST v5.0.0) (11). Mash (v2.2.1; Mash Screen) (12) was used for taxonomic assignment of assemblies based on the highest RefSeq (v93; https://www.ncbi.nlm.nih.gov/refseq) identities of >0.8 (excluding hits containing the words phage or plasmid) and cross-referencing to *V. diabolicus* classifications (13) (http://www.ncbi.nlm.nih.gov/genome). Analysis tools were used with default settings unless noted.

Isolate and genome details are listed in Table 1. Genome assemblies included 30 to 289 contigs and coverage depths of 29-fold to 317-fold. Total genome sizes ranged from 4.0 Mbp to 5.6 Mbp and contained 3,508 to 5,138 predicted genes. Taxonomic classification of the *de novo* genomes identified 48 V. alginolyticus, 15 *V. diabolicus*, 3 V. parahaemolyticus, 2 V. cholerae, 1 *V. metoecus*, 1 V. vulnificus, 1 *V. fluvialis*, and 4 unclassified *Vibrio* spp.

### Data availability.

Data for this study have been deposited in NCBI under BioProject PRJNA843722; raw reads and genome accession numbers are listed in Table 1.
